# Supplementary material for: Pandoravirus Celtis Illustrates the Microevolution Processes at Work in the Giant Pandoraviridae Genomes
Source: Front Microbiol. 2019 Mar 8;10:430. doi: 10.3389/fmicb.2019.00430 (PMC6418002; doi:10.3389/fmicb.2019.00430)
Supplement: Supplementary file 1 [file Data_Sheet_1.pdf]

Supplementary Table S1. Predicted non-coding RNAs (ncRNAs)

P. celtis gene #	Length (nt)	*P. quercus* homolog gene #	% identity	% overlap	BlastN E-value	Median RNAseq Read coverage
pclt_ncRNA_138	647	pqer_ncRNA_139	100	100	0	457
pclt_ncRNA_31	1432	pqer_ncRNA_42	100	100	0	453
pclt_ncRNA_137	1750	pqer_ncRNA_138	99.9	100	0	605
pclt_ncRNA_101	1041	pqer_ncRNA_104	99.8	100	0	420
pclt_ncRNA_92	585	pqer_ncRNA_95	98.1	100	0	567
pclt_ncRNA_149	968	pqer_ncRNA_145	97.9	100	0	569
pclt_ncRNA_146	1707	pqer_ncRNA_144	97.7	100	0	381
pclt_ncRNA_18	1416	pqer_ncRNA_24	96.9	100	0	389
pclt_ncRNA_60	1223	pqer_ncRNA_68	96.7	100	0	359
pclt_ncRNA_83	1259	pqer_ncRNA_82	95.1	100	0	226
pclt_ncRNA_28	1362	pqer_ncRNA_39	99.1	99.7	0	376
pclt_ncRNA_155	1997	pqer_ncRNA_149	99.8	99.6	0	733
pclt_ncRNA_144	648	pqer_ncRNA_142	96.9	99.5	0	717
pclt_ncRNA_109	2468	pqer_ncRNA_112	100	99.4	0	394
pclt_ncRNA_133	4456	pqer_ncRNA_134	97.2	99.3	0	354
pclt_ncRNA_150	2157	pqer_ncRNA_146	100	99.1	0	395
pclt_ncRNA_100	1363	pqer_ncRNA_103	100	99.0	0	995
pclt_ncRNA_65	813	pqer_ncRNA_70	100	99.0	0	253
pclt_ncRNA_30	735	pqer_ncRNA_40	97	98.9	0	324
pclt_ncRNA_153	1504	pqer_ncRNA_148	100	98.7	0	231
pclt_ncRNA_113	1576	pqer_ncRNA_117	94	98.7	0	540
pclt_ncRNA_77	854	pqer_ncRNA_80	100	98.6	0	475
pclt_ncRNA_16	1651	pqer_ncRNA_21	97.4	97.8	0	541
pclt_ncRNA_159	1587	pqer_ncRNA_153	97	97.8	0	263
pclt_ncRNA_4	1467	pqer_ncRNA_6	98.4	97.7	0	737
pclt_ncRNA_106	3617	pqer_ncRNA_109	98.1	97.6	0	336
pclt_ncRNA_5	1758	pqer_ncRNA_7	97.9	97.6	0	362
pclt_ncRNA_121	2235	pqer_ncRNA_127	97.1	96.6	0	358
pclt_ncRNA_24	2177	pqer_ncRNA_35	100	95.7	0	396
pclt_ncRNA_72	1652	pqer_ncRNA_73	99.4	95.0	0	283
pclt_ncRNA_34	1089	pqer_ncRNA_43	95.2	94.9	0	129
pclt_ncRNA_152	2018	pqer_ncRNA_147	100	94.9	0	299
pclt_ncRNA_41	1547	pqer_ncRNA_49	99.9	94.6	0	478
pclt_ncRNA_145	2158	pqer_ncRNA_143	95	94.3	0	142
pclt_ncRNA_13	2246	pqer_ncRNA_14	95.4	93.5	0	151
pclt_ncRNA_9	1912	pqer_ncRNA_11	99.8	93.4	0	388
pclt_ncRNA_98	1956	pqer_ncRNA_99	98	93.3	0	431
pclt_ncRNA_6	1281	pqer_ncRNA_8	99.4	92.3	0	260
pclt_ncRNA_143	1580	pqer_ncRNA_140	99.7	91.8	0	517
pclt_ncRNA_78	1415	pqer_ncRNA_81	100	91.8	0	268

pclt_ncRNA_7	659	pqer_ncRNA_9	96.3	91.0	0	279
pclt_ncRNA_10	961	pqer_ncRNA_13	96.2	90.9	0	304
pclt_ncRNA_90	1813	pqer_ncRNA_89	99.9	89.0	0	238
pclt_ncRNA_129	1198	pqer_ncRNA_131	100	87.2	0	404
pclt_ncRNA_91	921	pqer_ncRNA_91	100	86.3	0	292
pclt_ncRNA_97	1253	pqer_ncRNA_98	95.4	85.9	0	116
pclt_ncRNA_156	1369	pqer_ncRNA_150	99.1	85.8	0	211
pclt_ncRNA_53	873	pqer_ncRNA_62	99.6	85.3	0	524
pclt_ncRNA_125	907	pqer_ncRNA_130	95.2	84.8	0	402
pclt_ncRNA_161	1748	pqer_ncRNA_157	98.1	84.4	0	262
pclt_ncRNA_89	1706	pqer_ncRNA_88	98	84.3	0	588
pclt_ncRNA_116	1731	pqer_ncRNA_121	99.7	84.3	0	350
pclt_ncRNA_158	1329	pqer_ncRNA_151	100	84.0	0	218
pclt_ncRNA_124	1090	pqer_ncRNA_129	95.7	84.0	0	461
pclt_ncRNA_134	1155	pqer_ncRNA_135	99.9	83.3	0	791
pclt_ncRNA_43	1966	pqer_ncRNA_50	100	82.1	0	1041
pclt_ncRNA_20	556	pqer_ncRNA_28	99.1	82.0	0	733
pclt_ncRNA_102	1539	pqer_ncRNA_105	99.8	81.5	0	294
pclt_ncRNA_47	2129	pqer_ncRNA_54	99.7	78.6	0	219
pclt_ncRNA_123	1141	pqer_ncRNA_128	100	78.1	0	512
pclt_ncRNA_114	1287	pqer_ncRNA_117	69.8	76.6	$<10^{-119}$	117
pclt_ncRNA_59	2415	pqer_ncRNA_67	98	75.0	0	337
pclt_ncRNA_104	1835	pqer_ncRNA_107	99.9	74.7	0	261
pclt_ncRNA_112	1045	pqer_ncRNA_116	71.2	73.9	$<10^{-97}$	153
pclt_ncRNA_58	779	pqer_ncRNA_66	100	71.9	0	177
pclt_ncRNA_57	1335	pqer_ncRNA_65	96.9	71.6	0	770
pclt_ncRNA_54	1035	pqer_ncRNA_64	96.1	69.3	0	311
pclt_ncRNA_3	1728	pqer_ncRNA_5	98.6	69.0	0	771
pclt_ncRNA_74	1930	pqer_ncRNA_77	85.4	68.7	0	278
pclt_ncRNA_94	1478	pqer_ncRNA_96	99	60.7	0	655
pclt_ncRNA_15	1715	pqer_ncRNA_20	91	60.6	0	376
pclt_ncRNA_25	2986	pqer_ncRNA_37	100	59.2	0	142
pclt_ncRNA_17	1359	pqer_ncRNA_23	98.7	58.6	0	153
pclt_ncRNA_88	744	pqer_ncRNA_87	100	58.2	0	459
pclt_ncRNA_23	793	pqer_ncRNA_34	82.9	57.4	$<10^{-142}$	263
pclt_ncRNA_45	2238	pqer_ncRNA_52	99.9	53.4	0	219
pclt_ncRNA_67	1951	pqer_ncRNA_71	100	52.4	0	379
pclt_ncRNA_46	1930	pqer_ncRNA_53	100	50.9	0	213
pclt_ncRNA_105	2098	pqer_ncRNA_108	98.5	50.5	0	253
pclt_ncRNA_120	1630	pqer_ncRNA_124	95.1	49.9	0	304
pclt_ncRNA_14	1458	pqer_ncRNA_15	98.3	44.8	0	1608
pclt_ncRNA_70	1331	pqer_ncRNA_129	77.4	44.3	$<10^{-126}$	119
pclt_ncRNA_103	1921	pqer_ncRNA_106	97.2	29.7	0	531
pclt_ncRNA_36	2052	pqer_ncRNA_46	100	21.1	0	484
pclt_ncRNA_160	1507	pqer_ncRNA_153	74.3	20.2	$<10^{-51}$	230
pclt_ncRNA_128	1405	pqer_ncRNA_131	85.2	18.8	$<10^{-82}$	200

pclt_ncRNA_115	1384	pqer_ncRNA_118	83.9	18.8	$<10^{-73}$	276
pclt_ncRNA_95	1361	none				189
pclt_ncRNA_122	1565	none				144
pclt_ncRNA_69	1623	none				139
pclt_ncRNA_68	2508	none				207
pclt_ncRNA_147	1899	none				153
pclt_ncRNA_35	234	none				235
pclt_ncRNA_99	643	none				376
pclt_ncRNA_85	484	none				277
pclt_ncRNA_131	1933	none				247
pclt_ncRNA_162	325	none				587
pclt_ncRNA_62	399	none				301
pclt_ncRNA_108	808	none				136
pclt_ncRNA_55	568	none				183
pclt_ncRNA_130	593	none				466
pclt_ncRNA_29	3016	none				400
pclt_ncRNA_84	436	none				193
pclt_ncRNA_61	725	none				167
pclt_ncRNA_42	782	none				194
pclt_ncRNA_136	753	none				238
pclt_ncRNA_87	367	none				166
pclt_ncRNA_132	1582	none				357
pclt_ncRNA_107	1466	none				332
pclt_ncRNA_39	978	none				274
pclt_ncRNA_119	1477	none				390
pclt_ncRNA_49	750	none				230
pclt_ncRNA_117	1518	none				344
pclt_ncRNA_40	803	none				132
pclt_ncRNA_64	732	none				235
pclt_ncRNA_26	581	none				194
pclt_ncRNA_48	643	none				184
pclt_ncRNA_142	644	none				222
pclt_ncRNA_63	949	none				297
pclt_ncRNA_141	1593	none				674
pclt_ncRNA_96	1319	none				223
pclt_ncRNA_157	954	none				206
pclt_ncRNA_86	681	none				200
pclt_ncRNA_110	874	none				352
pclt_ncRNA_118	989	none				328
pclt_ncRNA_38	891	none				277
pclt_ncRNA_2	1683	none				271
pclt_ncRNA_11	727	none				92
pclt_ncRNA_82	1363	none				332
pclt_ncRNA_66	661	none				317
pclt_ncRNA_37	909	none				172
pclt_ncRNA_51	1389	none				310

pclt_ncRNA_44	915	none				343
pclt_ncRNA_139	1580	none				165
pclt_ncRNA_127	1071	none				262
pclt_ncRNA_12	1786	none				222
pclt_ncRNA_75	1435	none				218
pclt_ncRNA_22	903	none				189
pclt_ncRNA_71	1037	none				250
pclt_ncRNA_52	1249	none				160
pclt_ncRNA_126	935	none				207
pclt_ncRNA_140	1627	none				169
pclt_ncRNA_73	794	none				240
pclt_ncRNA_163	1992	none				236
pclt_ncRNA_80	1113	none				574
pclt_ncRNA_135	1174	none				196
pclt_ncRNA_111	1244	none				389
pclt_ncRNA_50	1550	none				172
pclt_ncRNA_154	1378	none				133
pclt_ncRNA_8	1095	none				319
pclt_ncRNA_27	2220	none				816
pclt_ncRNA_19	1935	none				278
pclt_ncRNA_151	1453	none				226
pclt_ncRNA_76	1863	none				130
pclt_ncRNA_56	2488	none				229
pclt_ncRNA_1	1646	none				391
pclt_ncRNA_21	2922	none				100
pclt_ncRNA_33	1821	none				659
pclt_ncRNA_81	2228	none				888
pclt_ncRNA_148	1849	none				355
pclt_ncRNA_79	2325	none				226

The predicted *P. celtis* ncRNA genes were compared to *P. quercus* genome sequence using BlastN. Previously annotated *P. quercus ncRNA* genes overlapping with each best matching genomic positions were defined as homologous (column 3) to the corresponding *P. celtis* ncRNA genes. The transcript size is given in column 2. The overlap size and % identity are given in columns 4 and 5 (if applicable). As all ncRNA homologs originate from two closely related genomes, strong sequence similarities may not imply the conservation of a function (if any). Most of ncRNAs are antisense to a protein-coding gene except for 7 of them (grey-filled rows) that are mostly intergenic. A little more than half (87/161=54%) of the predicted *P. celtis* ncRNA genes exhibit a homolog in *P. quercus*.

Supplementary Figures S1-S3

Figure S1.

TEM image of an ultrathin section of p. celtis and p. quercus viral particles. The structures of the particles of the other Pandoraviridae strains do not exhibit any noticeable difference.

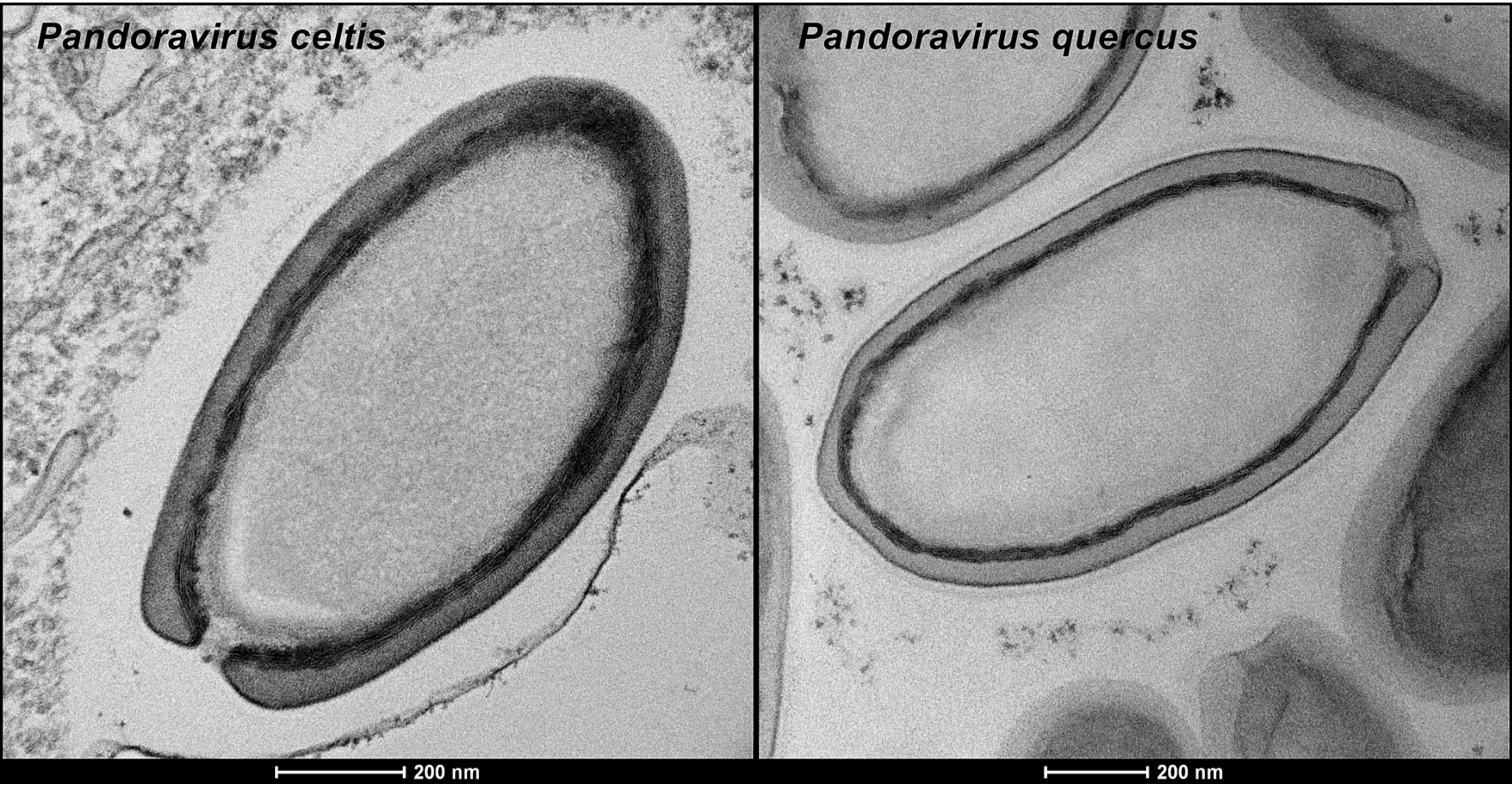
Pandoravirus celtis
Pandoravirus quercus
200 nm
200 nm

Figure S2.

Details of the p. celtis' hAT transposons. Details of the S0, S1, and S2 segments highlighted in Fig. 2. The genomic coordinates of the transposons in p. celtis (horizontal) and their homologous locations in p. quercus (vertical) are indicated. The cognate p. celtis' transposase gene name and its approximate location is indicated for each transposon. Other genes are not represented. Homologous segments are missing in p. quercus for S0 and S2 (See the Results section). The sequences of the transposon boundaries are shown above the dot-plots. The TIRs (Terminally Inverted Repeats) are highlighted in black. Conserved TIRs and TSDs (Target Site Duplications) are depicted using arrows.

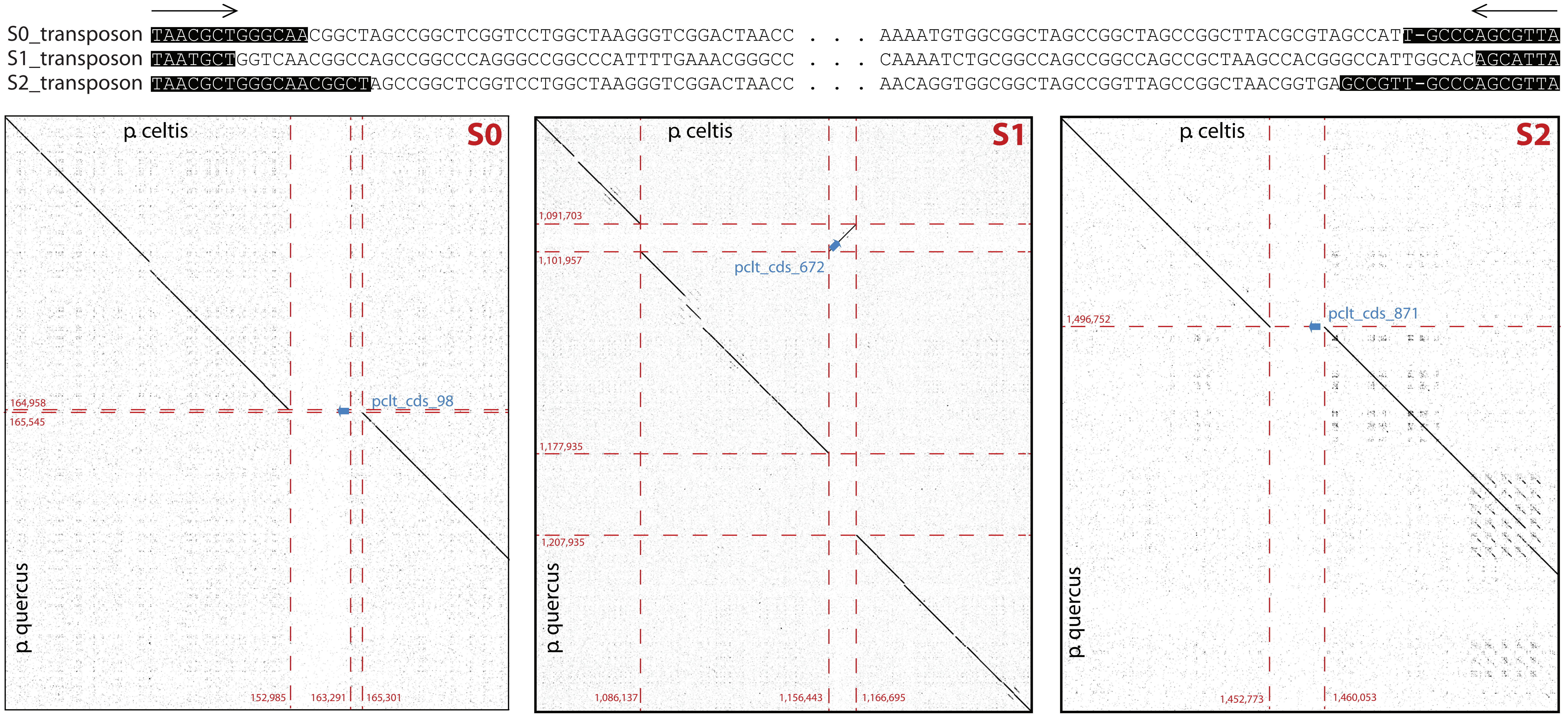
S0_transposon TAACGCTGGGCAACGGCTAGCCGGCTCGGTCCTGGCTAAGGGTCGGACTAACC . . . AAAATGTGGCGGCTAGCCGGCTAGCCGGCTTACGCGTAGCCATT-GCCCAGCGTTA
S1_transposon TAATGCTGGTCAACGGCCAGCCGGCCCAGGGCCGGCCCATTTTGAAACGGGCC . . . CAAAATCTGCGGCCAGCCGGCCAGCCGCTAAGCCACGGGCCATTGGCACAGCATTA
S2_transposon TAACGCTGGGCAACGGCTAGCCGGCTCGGTCCTGGCTAAGGGTCGGACTAACC . . . AACAGGTGGCGGCTAGCCGGTTAGCCGGCTAACGGTGAGCCGTT-GCCCAGCGTTA
p. celtis
S0
p. quercus
164,958
165,545
pclt_cds_98
152,985
163,291
165,301
p. celtis
S1
p. quercus
1,091,703
1,101,957
pclt_cds_672
1,177,935
1,207,935
1,086,137
1,156,443
1,166,695
p. celtis
S2
p. quercus
1,496,752
pclt_cds_871
1,452,773
1,460,053

Figure S3.

Details of two tandem repeat clusters of fascin-domain containing genes. See the Results section for comments. The dot-plot (horizontal: p. celtis, vertical: p. quercus) and the corresponding phylogenetic tree of the multiple paralogs illustrate the well-ordered dynamics of this highly repeated regions, alternating expansion, deletions and pseudogenization. For instance, we noticed the presence of two alleles of the pclt_886 proteins in our initial sequence data. The minor one (482 residues, used in this tree) is 96.7% identical to its pqer_868 homolog, while the major one results into a smaller protein (363 residues) diverging after the first 125 residues due to the presence of 4 indels in the rest of the gene. The evolutionary history of the fascin-domain containing proteins was inferred using the Neighbor-Joining method as implemented in Mega (Kumar S, et al. (2018) MEGA X: Molecular Evolutionary Genetics Analysis across computing platforms. Mol. Biol. Evol. 35:1547-1549.). The evolutionary distances were computed using the Poisson correction method. The analysis involved 31 amino acid sequences. All positions containing gaps and missing data were eliminated. There were 323 positions in the final dataset.

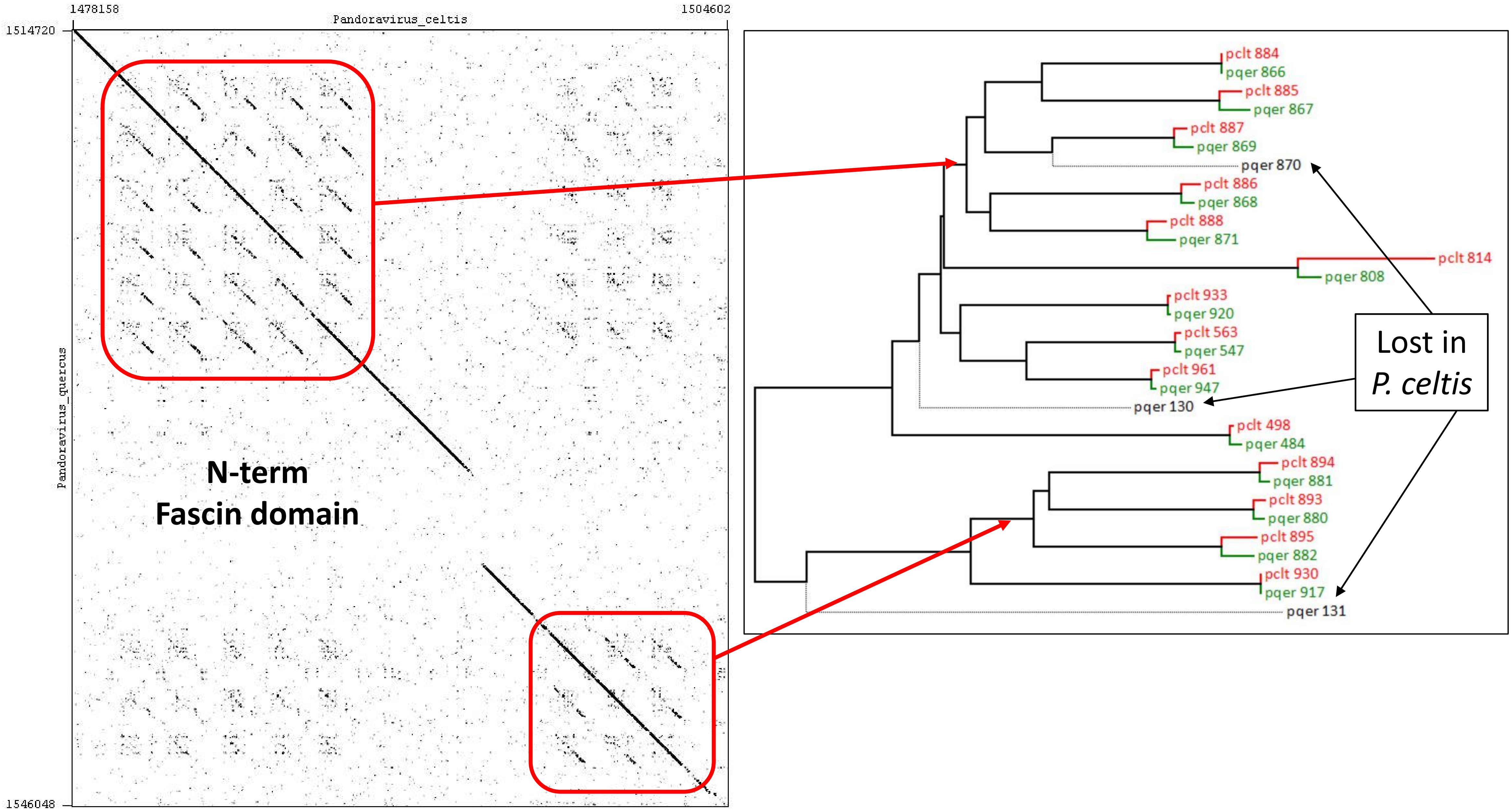
1478158
Pandoravirus_celtis
1504602
1514720
1546048
Pandoravirus_quercus
N-term
Fascin domain
pclt 884
pqer 866
pclt 885
pqer 867
pclt 887
pqer 869
pqer 870
pclt 886
pqer 868
pclt 888
pqer 871
pclt 814
pqer 808
pclt 933
pqer 920
pclt 563
pqer 547
pclt 961
pqer 947
pqer 130
pclt 498
pqer 484
pclt 894
pqer 881
pclt 893
pqer 880
pclt 895
pqer 882
pclt 930
pqer 917
pqer 131
Lost in
P. celtis
